# Co-deposition of SOD1, TDP-43 and p62 proteinopathies in ALS: evidence for multifaceted pathways underlying neurodegeneration

**DOI:** 10.1186/s40478-022-01421-9

**Published:** 2022-08-25

**Authors:** Benjamin G. Trist, Jennifer A. Fifita, Alison Hogan, Natalie Grima, Bradley Smith, Claire Troakes, Caroline Vance, Christopher Shaw, Safa Al-Sarraj, Ian P. Blair, Kay L. Double

**Affiliations:** 1 Brain and Mind Centre and School of Medical Sciences (Neuroscience), Faculty of Medicine and Health, The University of Sydney, Sydney, NSW 2006 Australia; 2 Centre for Motor Neuron Disease Research, Macquarie Medical School, Faculty of Medicine, Health and Human Sciences, Macquarie University, Sydney, NSW Australia; 3 Maurice Wohl Clinical Neuroscience Institute and the Institute of Psychiatry, Psychology and Neuroscience, King’s College London, Camberwell, London, SE5 9RT UK; 4 London Neurodegenerative Diseases Brain Bank, Department of Basic and Clinical Neuroscience, Institute of Psychiatry, Psychology and Neuroscience, King’s College London, London, SE5 8AF UK

**Keywords:** Superoxide dismutase-1, TAR DNA-binding protein 43, p62/SQSTM1, Proteinopathy, Amyotrophic lateral sclerosis

## Abstract

Multiple neurotoxic proteinopathies co-exist within vulnerable neuronal populations in all major neurodegenerative diseases. Interactions between these pathologies may modulate disease progression, suggesting they may constitute targets for disease-modifying treatments aiming to slow or halt neurodegeneration. Pairwise interactions between superoxide dismutase 1 (SOD1), TAR DNA-binding protein 43 (TDP-43) and ubiquitin-binding protein 62/sequestosome 1 (p62) proteinopathies have been reported in multiple transgenic cellular and animal models of amyotrophic lateral sclerosis (ALS), however corresponding examination of these relationships in patient tissues is lacking. Further, the coalescence of all three proteinopathies has not been studied in vitro or in vivo to date. These data are essential to guide therapeutic development and enhance the translation of relevant therapies into the clinic. Our group recently profiled SOD1 proteinopathy in post-mortem spinal cord tissues from familial and sporadic ALS cases, demonstrating an abundance of structurally-disordered (dis)SOD1 conformers which become mislocalized within these vulnerable neurons compared with those of aged controls. To explore any relationships between this, and other, ALS-linked proteinopathies, we profiled TDP-43 and p62 within spinal cord motor neurons of the same post-mortem tissue cohort using multiplexed immunofluorescence and immunohistochemistry. We identified distinct patterns of SOD1, TDP43 and p62 co-deposition and subcellular mislocalization between motor neurons of familial and sporadic ALS cases, which we primarily attribute to *SOD1* gene status. Our data demonstrate co-deposition of p62 with mutant and wild-type disSOD1 and phosphorylated TDP-43 in familial and sporadic ALS spinal cord motor neurons, consistent with attempts by p62 to mitigate SOD1 and TDP-43 deposition. Wild-type SOD1 and TDP-43 co-deposition was also frequently observed in ALS cases lacking *SOD1* mutations. Finally, alterations to the subcellular localization of the three proteins were tightly correlated, suggesting close relationships between the regulatory mechanisms governing the subcellular compartmentalization of these proteins. Our study is the first to profile spatial relationships between SOD1, TDP-43 and p62 pathologies in post-mortem spinal cord motor neurons of ALS patients, previously only studied in vitro. Our findings suggest interactions between these three key ALS-linked proteins are likely to modulate the formation of their respective proteinopathies, and perhaps the rate of motor neuron degeneration, in ALS patients.

**Supplementary Information:**

The online version contains supplementary material available at 10.1186/s40478-022-01421-9.

## Introduction

Intracellular mislocalization and deposition of key proteins is a neuropathological hallmark of all neurodegenerative diseases, and these processes are believed to contribute to neuron death within vulnerable regions of the brain and spinal cord in these disorders [42]. Research in this field has traditionally focussed on individual proteins known to form characteristic pathologies in each disorder, such as α-synuclein in Parkinson’s disease, β-amyloid or tau in Alzheimer’s, TAR DNA-binding protein 43 (TDP-43) or superoxide dismutase 1 (SOD1) in amyotrophic lateral sclerosis. Increasingly however, multiple types of proteinopathy have been demonstrated within vulnerable neurons in these disorders, sparking interest in potential synergies between these pathologies and their contribution to disease pathogenesis [43]. Biochemical mechanisms underlying the interaction and co-deposition of these proteins are currently poorly understood, with available data primarily derived from in vitro and in vivo models which are not able to accurately recapitulate complex aggregation proteomes observed in patient brains. Whilst these investigations provide important clues about potential mechanisms by which two proteins may interact, it is essential to conduct parallel investigations in patient tissues to confirm whether interactions identified in model systems actually occur in the human central nervous system. We are most likely to advance our understanding of the key drivers of neurodegeneration in these disorders by combining data from both avenues of investigation, including identifying promising therapeutic targets in the process.

Pairwise interactions between SOD1, TDP-43 and ubiquitin-binding protein 62/sequestosome 1 (p62) proteinopathies have been reported in multiple transgenic cellular and animal models of amyotrophic lateral sclerosis (ALS), however patient data is lacking. SOD1 is an important antioxidant protein, and was the first dominantly-inherited genetic risk factor for ALS [40]. Mutations to the *SOD1* gene, together with atypical post-translational modification of SOD1 protein, promote misfolding, dysfunction and deposition of the protein, which are collectively strongly implicated in motor neuron degeneration in this disorder [48]. TDP-43 is an essential DNA/RNA binding protein that regulates transcription, alternative splicing and mRNA stability, and is predominantly localized within the nucleus in healthy neurons [12]. In ALS TDP-43 undergoes extensive atypical post-translational modification, resulting in its nucleocytoplasmic mislocalization, cytoplasmic accumulation and aggregation [44]. Pathological TDP-43 and SOD1 exhibit cross-seeding behaviours in cultured neurons [36, 50], however are rarely identified within the same spinal cord motor neurons in post-mortem tissues from ALS patients, questioning whether interactions identified in vitro are translatable to human ALS pathogenesis. Another ALS hallmark protein, p62, regulates autophagy and the shuttling of ubiquitinated proteins for degradation by the ubiquitin–proteasome system (UPS) in healthy neurons [24]. p62 is a reliable component of cytoplasmic protein inclusions in spinal cord motor neurons of sporadic (s)ALS cases [33]. p62 directly interacts with mutant, but not wild-type, SOD1 and is reported to enhance formation of non-toxic mutant SOD1 aggregates in cellular and murine models of *SOD1*-linked (*SOD1-*)fALS [17], however corresponding data from *SOD1*-fALS patients is lacking. p62 also mitigates TDP-43 aggregation in an autophagy- and proteasome-dependent manner in cultured neuronal progenitors [5], with co-deposition of these proteins reported in post-mortem spinal cord tissues from sALS patients. Importantly, the coalescence of SOD1, TDP-43 and p62 proteinopathies has not been assessed in vitro or in vivo to date, and may inform on novel therapeutic approaches capable of slowing or halting the development of multiple damaging co-pathologies.

Our group recently reported the accumulation and mislocalization of structurally-disordered (dis)SOD1 protein in spinal cord motor neurons of *SOD1*-fALS, non-*SOD1*-linked (non-*SOD1*-)fALS and sALS cases [47]. To examine relationships between this, and other, ALS-linked proteinopathies, we constructed overlapping spatial profiles of disSOD1, TDP-43 and p62 proteinopathies in post-mortem spinal cord tissues using a combination of DAB immunohistochemistry and multiplexed immunofluorescence microscopy. Our data reveal distinct SOD1, TDP-43 and p62 aggregation profiles within motor neurons of *SOD1*-fALS, non-*SOD1*-fALS and sALS cases. We demonstrate close spatial relationships between disordered SOD1, pathological TDP-43 and p62 pathologies within these neurons, suggesting interactions between these proteins modulate the formation of their respective proteinopathies.

## Methods

### Human post-mortem tissues

Post-mortem tissue cases utilized in this study are the same as those employed in our related study examining disSOD1 pathology and post-translational modification in ALS [47]. Formalin-fixed and fresh-frozen human post-mortem spinal cord tissues from patients with *SOD1*-associated familial amyotrophic lateral sclerosis (*SOD1*-fALS; *n* = 3), non-*SOD1*-associated fALS (non-*SOD1*-fALS; *n* = 4), sporadic ALS (sALS; *n* = 9) and age-matched controls (*n* = 10) were obtained from the MRC London Neurodegenerative Diseases Brain Bank (King’s College, London, UK) and the University of Maryland Brain and Tissue Bank, a biorepository of the NIH NeuroBioBank (Maryland, USA). Power analyses of data describing the proportion of motor neurons exhibiting granular SOD1, nuclear TDP-43 or p62 pathology in the ventral spinal cord of sALS cases (*n* = 7) compared with age-matched controls (*n* = 9) demonstrated that an *n* of 7 cases per diagnostic group was strongly powered to detect differences in pathological features of interest (100% power, two-tailed *t-*test, α = 5%; SPSS software, IBM, Armonk, NY, USA). Sample sizes were limited for *SOD1*-fALS (*n* = 3) and non-*SOD1*-fALS (*n* = 4) ALS subgroups due to the rarity of these tissues, however retrospective power analyses demonstrated our study was strongly powered to detect differences in these tissues compared with age-matched controls due to large effect sizes observed for these variables (99.9–100% power for data describing the proportion of motor neurons exhibiting granular SOD1, nuclear TDP-43 or p62 pathology). Clinical diagnoses of ALS were determined using patient histories received from the donors' physicians, and pathological identification of spinal cord motor neuron loss by brain bank neuropathologists confirmed clinical diagnoses. All ALS cases were free of other neurological or neuropathological conditions. Age-matched control cases were free of any clinically diagnosed neurological disorders and neuropathological abnormalities. Demographic and clinical information for all cases are detailed in Additional file 1: Table S1. We were unable to statistically evaluate whether sex influenced any measured variables due to small *n*’s. Neither age nor post-mortem interval were significantly correlated with any measured variables of interest in this study.

Formalin-fixed tissues were obtained from the cervical and thoracic spinal cord of all cases for immunohistochemical analyses, and fresh-frozen tissue obtained from the occipital cortex for *SOD1* and *C9ORF72* genotyping. Formalin-fixed tissues were embedded in paraffin and 7 µm sections prepared for immunohistochemical analyses. Post-mortem cases were randomly numbered by a secondary investigator prior to experimentation to blind primary investigators to case diagnoses.

### SOD1 and C9ORF72 genotyping

*SOD1* and *C9ORF72* genotyping was performed in our post-mortem ALS and control cases as previously described [28]. DNA extraction from human brain tissue was performed using the DNeasy DNA extraction kit (Cat. #69506; Qiagen, Hilden, Germany), according to manufacturer’s instructions. All five exons of *SOD1*, together with greater than 10 bp of flanking sequence, were sequenced using PCR amplification and Sanger sequencing. The C9orf72 hexanucleotide repeat sequence was amplified using the repeat primed PCR method, and was subsequently analysed by fragment analysis [28]. Pathogenic status of the *C9ORF72* gene was defined as greater than 30 repeats of the hexanucleotide GGGGCC expansion within the first intron of the C9ORF72 gene [37]. All results were independently analysed by two team members. Inconclusive samples were repeated until a conclusive result was obtained. Positive samples were repeated twice to confirm results. Mutations in the *SOD1* gene were identified in ALS cases 11 (I113T), 12 (I113T) and 13 (D101G), whereas hexanucleotide repeat expansions in the *C9ORF72* gene were identified in ALS cases 14, 16 and 17 (Additional file 1: Table S1).

### Immunohistochemistry

Seven-micron fixed tissue sections used for 3,3′-Diaminobenzidine (DAB) staining and brightfield microscopy were deparaffinated using xylene and graded ethanol. Antigen retrieval was performed in citrate buffer (pH 6; Fronine, Australia) at 95 °C for 30 min. Sections were washed with 50% ethanol, incubated with 3% H_2_O_2_ (Fronine, Australia) in 50% ethanol to quench non-specific peroxidase activity, and blocked in 0.5% casein, 1% bovine serum albumin diluted in PBS for 1.5 h at room temperature. Sections were incubated overnight with appropriate primary antibodies diluted in blocking solution (Additional file 1: Table S3), and primary antibodies detected using biotinylated IgG secondary antibodies (2 h, room temperature, 1:200 diluted in blocking solution) (Vector Laboratories, Burlingame, CA) followed by Vector Elite Kit tertiary antibody complex (2 h, room temperature, 1:100 diluted in PBS) (Vector Laboratories, Burlingame, CA). Antibody binding was visualised using a DAB (Sigma) solution containing cobalt and nickel ions, changing the chromogen colour from brown to black. Sections were finally dehydrated using graded ethanol and xylene, and mounted with DPX. Staining was visualized and imaged using an Olympus VS 120 Slide Scanner at 40 × magnification (Olympus-life science, Shinjuku, Tokyo, Japan), and images processed using OlyVIA (Version 3.1; Olympus-life science) or Fiji (National Institute of Health (NIH)) software. A *no primary* negative control was also included for each diagnostic group to identify protocol- and tissue-related artefacts independent of primary antibody immunoreactivity (Additional file 1: Figure S1), involving tissues processed as above but in the absence of primary antibodies.

### Quantification of DAB-immunostained pathological features of interest

Immunostaining for p62 and TDP-43 was performed in ventral horn grey matter of the cervical and thoracic spinal cord as above, using appropriate primary antibodies (Additional file 1: Table S3). TDP-43 immunostaining utilized antibodies recognizing a phosphorylated C-terminal fragment of TDP-43, commonly implicated in TDP-43 deposition (pathological TDP-43; pTDP-43), as well as non-phosphorylated TDP-43. Pathologies of interest were intracellular pTDP-43 deposits, diffuse cytosolic pTDP-43, nuclear TDP-43, intracellular p62 deposits, diffuse cytosolic p62, and nuclear p62. Motor neurons were considered to be negative for a given pathology of interest if they exhibited no immunoreactivity for that pathology, e.g. if no nuclear TDP-43 immunoreactivity was present then that motor neuron was counted as nuclear TDP-43 negative. Counts of each pathology were expressed as the proportion of neurons exhibiting evidence of that pathology to account for differences in motor neuron number between cervical and thoracic spinal cord levels. The number of cells counted to quantify each pathology of interest are presented in (Additional file 1: Table S4).

### Multiplexed immunofluorescence

Fluorescent staining and confocal microscopy employed 7 µm fixed tissue sections, which were deparaffinated, antigen retrieved, quenched, blocked and incubated with appropriate primary antibodies (Additional file 1: Table S3) as above. Multiplexed fluorescent immunolabelling was performed using OPAL fluorophores and spectral DAPI (Akoya Biosciences, Menlo Park, CA, USA) according to the manufacturer’s instructions. Antibodies were stripped in between OPAL fluorophore incubations by incubating sections in AR6 commercial buffer (Akoya Biosciences, USA) at 95 °C for 40 min. After fluorophore incubations sections were washed with PBS and cover-slipped with 80% glycerol. Images were collected at 60 × magnification using a Nikon C2 + Confocal Microscope System (Nikon, Minato City, Tokyo, Japan) and Nikon NIS-elements software (Version 5.20.02; Nikon, Japan), and were viewed and analysed using Fiji software (National Institute of Health, Bethesda, Maryland, USA).

As above, a *no primary* negative control was included for each diagnostic group to identify non-specific fluorescent signals originating from autofluorescent tissue features (e.g. lipofuscin) or direct binding of secondary antibodies to tissues (Additional file 1: Figure S1). During acquisition of double- or triple-immunolabelled fluorescent images a sequential protocol was used where each fluorophore was excited and the corresponding channel individually acquired, before moving onto the next fluorophore and channel. This minimized spectral cross-contamination between OPAL650, OPAL620 and OPAL520 fluorophores (Additional file 1: Figure S1).

### Quantification of SOD1, TDP-43 and p62 pathology colocalization

Ten spinal cord tissue sections from each diagnostic group, including at least one section from all available cases, were immunostained for disordered SOD1, pTDP-43 and p62, and cell nuclei counterstained with DAPI. We then imaged all motor neurons within these sections exhibiting evidence of disordered SOD1, pTDP-43 or p62 neuropathological abnormalities. We imaged a total of 84, 104 and 123 motor neurons in *SOD1*-fALS, non-*SOD1*-fALS and sALS cases, respectively (Additional file 1: Table S4). Where multiple pathologies were present within an imaged motor neuron, we also noted whether there was spatial overlap (colocalization) or whether the pathologies were coincidental within an individual neuron. To reconcile differences in the total number of motor neurons imaged for each group, quantitative data on colocalization or coincidence of the three pathologies was expressed as the proportion of imaged motor neurons exhibiting evidence of that condition.

### Statistical analyses

Statistical analyses were performed using PASW v18 (SPSS inc., Chicago, IL, USA). Parametric tests or descriptive statistics with parametric assumptions (one-way ANOVA, Pearson's r) were used for variables (proportions of motor neurons with pTDP-43 pathology or nuclear TDP-43) meeting the associated assumptions. Non-parametric tests or statistics (Kruskal–Wallis H test, Spearman's r) were used for variables (proportions of motor neurons with p62 pathology or nuclear p62) where the observed data did not fit the assumptions of parametric tests. Data normality was assessed using the D'Agostino-Pearson and Shapiro–Wilk normality tests, whereby both tests needed to be passed for data to meet the assumption of a normal distribution. Outliers were identified using the combined robust regression and outlier removal (ROUT) method with a maximum false discovery rate of 5%. Final values were reported as mean ± SEM. A *p* value of less than 0.05 was accepted as the level of significance.

## Results

### Nucleocytoplasmic TDP-43 mislocalization and aggregation are associated with disSOD1 pathology in post-mortem ALS ventral spinal cord

**Fig. 1:**
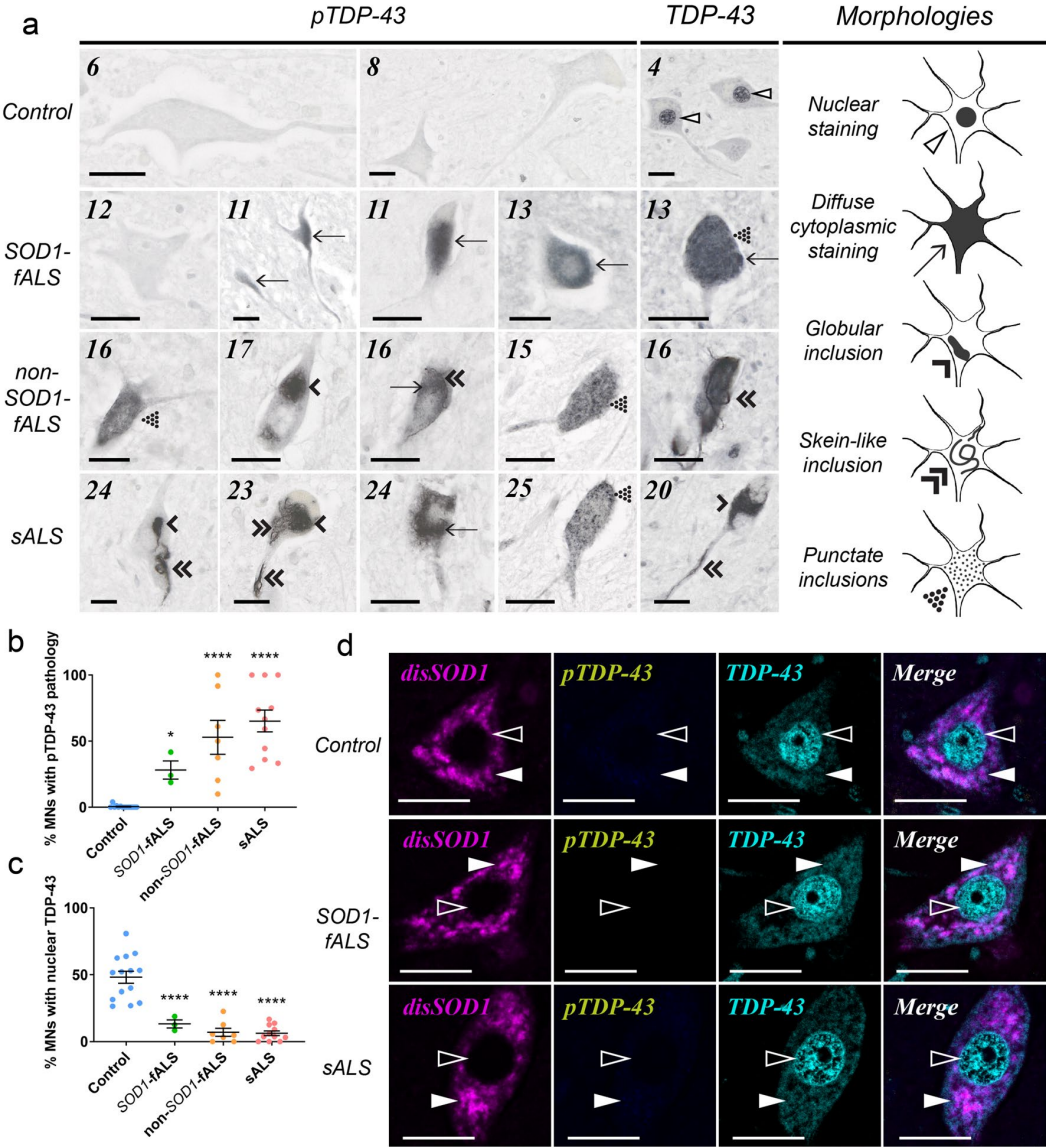
TDP-43 cytoplasmic mislocalization and deposition in the vulnerable ventral spinal cord of ALS cases. **a** Both pan and phosphorylated (p)TDP-43 antibodies identified diffuse cytoplasmic TDP-43 staining (arrows), as well as punctate (dotted arrowheads), globular (single downwards arrowheads) and fibrillar skein-like (double downwards arrowheads) TDP-43-immunopositive inclusions, in ventral spinal cord motor neurons of all ALS cases and a small proportion of controls. Pan TDP-43 immunostaining also revealed nuclear TDP-43 (open arrowheads) in ventral spinal cord motor neurons of ALS cases and controls. Case numbers (Additional file 1: Table S1**)** are listed in the top left corner of each panel. **b**, **c** The proportion of motor neurons possessing diffuse cytoplasmic TDP-43 or cytoplasmic TDP-43 inclusions (collectively termed TDP-43 pathology) was higher in ALS cases compared with controls (**b**; One-way ANOVA with Holm-Sidak’s multiple comparisons post-hoc tests; *SOD1*-fALS: *p* = 0.05; non-*SOD1*-fALS: *p* < 0.0001; sALS: *p* < 0.0001), whereas the proportion of motor neurons exhibiting nuclear TDP-43 immunostaining was significantly reduced in ALS cases compared with controls (**c**; One-way ANOVA with Holm-Sidak’s multiple comparisons post-hoc tests, *p* < 0.0001 for all comparisons). Data in **b** and **c** represent mean ± SEM. *p < 0.05, ****p < 0.0001. **d** Phosphorylated (p)TDP-43 pathology is absent within control, *SOD1*-fALS and sALS spinal cord motor neurons exhibiting non-pathological nuclear TDP-43 (open arrowheads) and granular SOD1 immunostaining (closed arrowheads). Scale bars in **a** and **d** represent 25 µm. Antibody details are listed in Additional file 1: Table S3. No immunostaining was observed in spinal cord tissue sections processed as above in the absence of primary antibodies (Additional file 1: Figure S1)

Phosphorylation and C-terminal fragmentation of TDP-43 are central to its pathological aggregation in vitro and in vivo [4, 21], and phosphorylated TDP-43 is heavily enriched in TDP-43 aggregates in post-mortem spinal cord tissues from ALS cases [21]. Using a monoclonal TDP-43 antibody specifically recognizing phosphorylated, C-terminal TDP-43 (pTDP-43), we identified diffuse cytoplasmic pTDP-43 immunoreactivity within spinal cord motor neurons of all ALS cases, but only three-of-nine controls (Fig. 1a, Table 1). Cytoplasmic pTDP-43-immunopositive motor neuron inclusions of varying morphologies were also present within three-of-four non-*SOD1*-fALS cases, all seven sALS cases, and three-of-nine control cases, but were absent within *SOD1*-fALS cases (Fig. 1a, Table 1). The proportion of motor neurons exhibiting either form of pTDP-43 pathology was negligible in control cases (0.4%) compared with all ALS subgroups (24–58%; Fig. 1b, Table 1).

**Table 1:** Characterization and quantification of pTDP-43-immunopositive spinal cord motor neuron (MN) inclusions, as well as diffuse cytoplasmic pTDP-43 staining and nuclear pan TDP-43 immunoreactivity, in seven micron paraffin-embedded formalin-fixed tissue sections of the cervical and thoracic spinal cord from all post-mortem tissue cases

Case #	Diagnostic group	pTDP-43 morphology				Quantification (%MNs)	
		Globular	Skein-like	Punctate	Diffuse	Incl./diff	Nuclear
1	Age-matched control	−	−	−	−	−	+++
2	Age-matched control	NA	NA	NA	NA	NA	NA
3	Age-matched control	−	−	−	−	−	+++
4	Age-matched control	√	−	−	−	+	++
5	Age-matched control	−	−	−	−	−	+++
6	Age-matched control	√	−	−	−	+	+++
7	Age-matched control	−	−	−	−	−	+++
8	Age-matched control	−	−	−	−	−	++
9	Age-matched control	−	−	−	−	−	++
10	Age-matched control	√	−	−	−	+	++
11	fALS (SOD1, I113T)	−	−	−	√	+	+
12	fALS (SOD1, I113T)	−	−	−	√	++	+
13	fALS (SOD1, D101G)	−	−	−	√	+	+
14	fALS (C9ORF72, 30+positive)	−	−	−	√	++	+
15	fALS (unknown mutation)	√	√	√	√	++	+
16	fALS (C9ORF72, 30+positive)	√	√	√	√	++++	+
17	fALS (C9ORF72, 30+positive)	√	√	−	√	+++	+
18	sALS	√	−	−	√	+++	+
19	sALS	√	√	−	√	++++	+
20	sALS	√	√	√	√	++++	+
21	sALS	√	√	−	√	++	+
22	sALS	NA	NA	NA	NA	NA	NA
23	sALS	√	√	√	√	+++	+
24	sALS	√	√	√	√	++++	+
25	sALS	√	√	√	√	++	+
26	sALS	NA	NA	NA	NA	NA	NA

Fixed tissues were not available (NA) for some cases. The four morphologies of pTDP-43 pathology were noted as present (√) or absent (−), and the proportion of motor neurons exhibiting any of the four morphologies quantified. The proportion of motor neurons exhibiting nuclear pan TDP-43 immunostaining was also quantified. Quantification classifications;− = 0%, + =  > 0–25%, ++  = 26–50%, +++ = 51–75%, ++++ = 76–100%

**Fig. 2:**
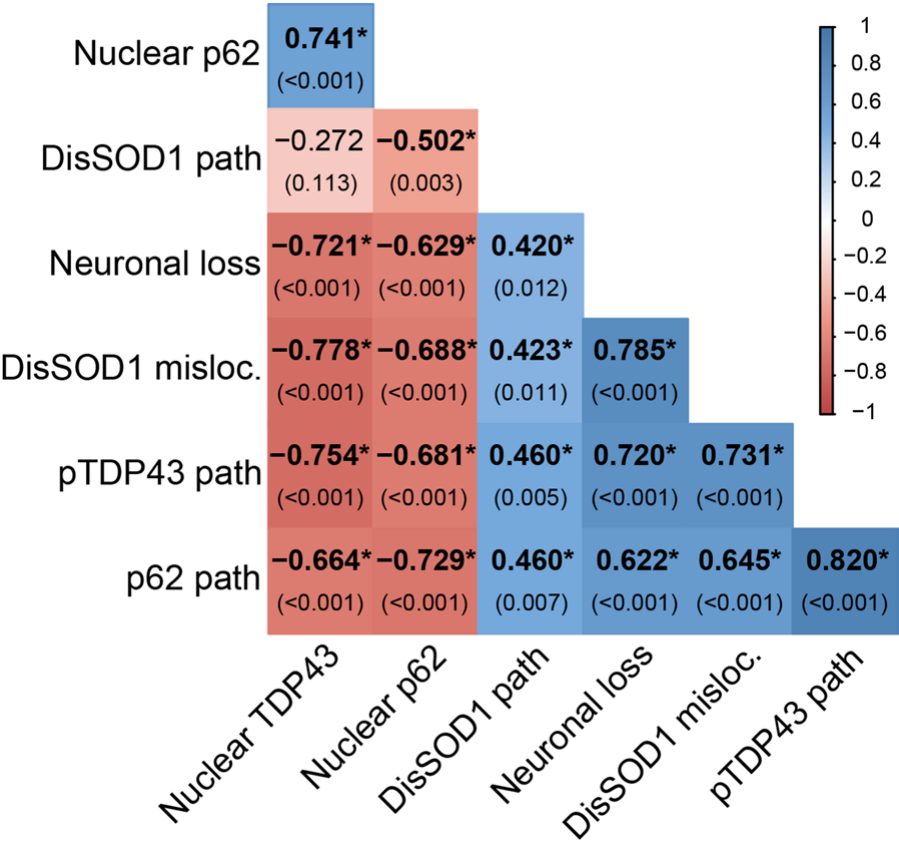
Neuropathological correlates of SOD1, TDP43 and p62 proteinopathies in spinal cord motor neurons of post-mortem ALS and control cases. Quantitative data describing disordered SOD1 (DisSOD1) pathology, neuronal loss and the proportion of motor neurons exhibiting DisSOD1 mislocalization in the spinal cord of our post-mortem tissue cohort are published in a related article [47]. Spearman’s *r* coefficient (top) and the *p* value (bottom) of each correlation are stated within panel. Positive and negative Spearman’s r coefficients are delineated by a binary colour gradient. *n* = 33–35 data points for each correlation. A correlation is considered to be strong if Spearman’s *r* = 0.5 or higher. Asterisks indicate statistically significant correlations

The development of cytoplasmic TDP-43 pathology is thought to be driven by nucleocytoplasmic TDP-43 mislocalization in cellular and animal models of ALS [44]. We utilized a polyclonal pan TDP-43 antibody to assess the subcellular localization of full-length TDP-43 in ALS and control spinal cord motor neurons, revealing a 6.5-fold reduction in the proportion of motor neurons possessing nuclear TDP-43 in all ALS subgroups compared with controls (range: 3.7-to-9.5-fold; Fig. 1a, c, Table 1). A reduction in the proportion of motor neurons possessing nuclear TDP-43 was strongly correlated with a higher proportion of motor neurons exhibiting cytoplasmic pTDP-43 pathology (Fig. 2), reinforcing existing data demonstrating a close interdependence between TDP-43 phosphorylation, mislocalization and aggregation in spinal cord motor neurons in ALS [34]. A greater proportion of motor neurons exhibiting nuclear TDP-43 export and cytoplasmic pTDP-43 pathology was also correlated with more severe motor neuron degeneration (Fig. 2), supporting a role for these changes in ALS pathogenesis.

Strong positive correlations were identifed between the proportion of motor neurons exhibiting cytoplasmic pTDP-43 pathology or nuclear TDP-43 depletion, and disSOD1 mislocalization (Fig. 2) [47], suggesting regulatory pathways governing the subcellular compartmentalization of these two proteins are either shared or closely related. Co-localization immunofluorescence reinforced this relationship, confirming the absence of cytoplasmic disSOD1 or pTDP-43 pathology within motor neurons exhibiting non-pathological nuclear TDP-43 and granular SOD1 immunostaining (Fig. 1d). On the contrary, a correlation was also observed between the proportion of motor neurons exhibiting cytoplasmic disSOD1 and pTDP-43 pathology (Fig. 2), suggesting common factors driving the deposition of these proteins.

### Cytoplasmic p62 accumulation and deposition is associated with ALS-linked pathologies in the degenerating ALS spinal cord

**Fig. 3:**
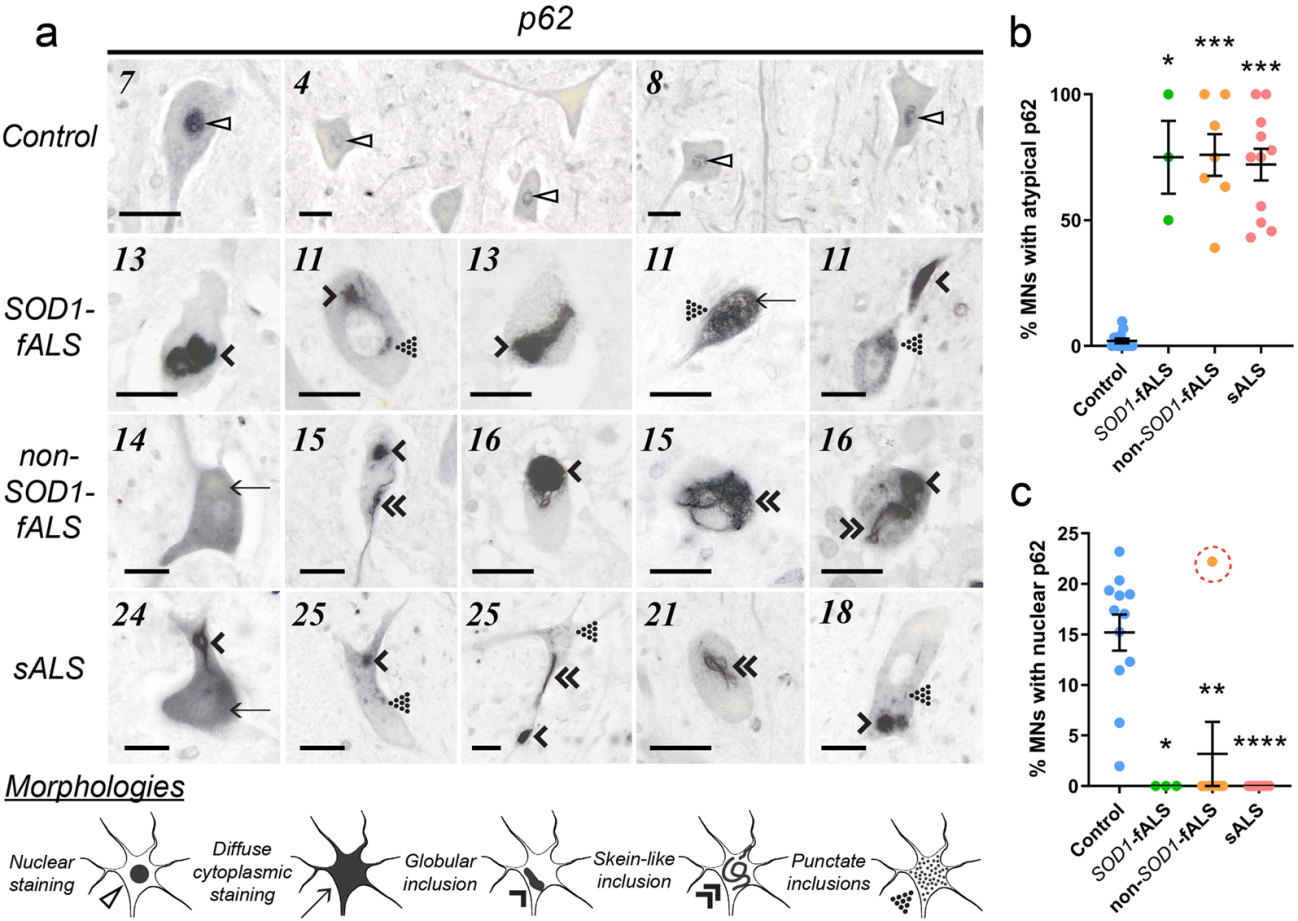
Altered p62 localization and deposition in the vulnerable ventral spinal cord of ALS cases. a p62 immunostaining revealed nuclear p62 (open arrowheads) in ventral spinal cord motor neurons of one ALS case and all controls. Diffuse cytoplasmic p62 staining (arrows), as well as punctate (dotted arrowheads), globular (single downwards arrowheads) and fibrillar skein-like (double downwards arrowheads) p62-immunopositive inclusions, were also identified in ventral spinal cord motor neurons of all ALS cases and a small proportion of controls. Case numbers (Additional file 1: Table S1) are listed in the top left corner of each panel and scale bars represent 25 µm. Antibody details are listed in Supplementary Table 3. No immunostaining was observed in spinal cord tissue sections processed as above in the absence of primary antibodies (Additional file 1: Figure S1). b The proportion of motor neurons exhibiting diffuse cytoplasmic p62 or cytoplasmic p62 inclusions (collectively termed atypical p62) was higher in ALS cases compared with controls (Kruskal–Wallis H test with Dunn’s multiple comparisons post-hoc tests; *SOD1*-fALS: *p* = 0.018; non-*SOD1*-fALS: *p* = 0.0006; sALS: *p* = 0.0002). **c** In contrast, nuclear p62 immunostaining was absent in all but one ALS case (open red circle), resulting in a significant reduction in the proportion of motor neurons exhibiting nuclear p62 compared with controls (Kruskal–Wallis H test with Dunn’s multiple comparisons post-hoc tests; *SOD1*-fALS: *p* = 0.01; non-*SOD1*-fALS: *p* = 0.004; sALS: *p* < 0.0001). Data in b and c represent mean ± SEM. *p < 0.05, **p < 0.01, ***p < 0.001, ****p < 0.0001

p62-immunoreactive motor neuron inclusions are a reliable hallmark of sALS in post-mortem patient spinal cord tissues [33], however the presence of this pathology in SOD1- and non-SOD1-fALS cases is less well understood. Immunostaining of post-mortem spinal cord tissues for p62 revealed inclusions of varying morphologies within 42–56% of motor neurons in all ALS cases, but only 4% of motor neurons in one-of-seven controls (Fig. 3a, Table 2). In addition to inclusions, strong diffuse cytoplasmic p62 immunostaining was observed within 36–63% of motor neurons in one-of-three *SOD1*-fALS cases, all four non-*SOD1*-fALS cases and five-of-seven sALS cases, but only 4% of motor neurons in three-of-nine control cases (Fig. 3a, Table 2). Collectively, these data demonstrate that cytoplasmic p62 accumulation and deposition are a common (72–76%) feature of all types of ALS, but are rarely present in healthy controls (2%; Fig. 3a, b, Table 2). These data are consistent with the accepted physiological function of this protein in sequestering toxic misfolded proteins into non-toxic cytoplasmic protein aggregates for clearance by autophagy or the UPS [17]; it is likely that such aggregates accumulate over time as disease severity increases and downstream proteostatic pathways become increasingly dysfunctional. Higher proportions of motor neurons exhibiting nuclear p62 depletion or cytoplasmic p62 pathology were indeed correlated with higher proportions of motor neurons possessing disSOD1 or pTDP-43 pathologies (Fig. 2).

**Table 2:** Characterization and quantification of p62-immunopositive spinal cord motor neuron (MN) inclusions, as well as diffuse cytoplasmic and nuclear p62 staining, in seven micron paraffin-embedded formalin-fixed tissue sections of the cervical and thoracic spinal cord from all post-mortem tissue cases

Case #	Diagnostic group	p62 morphology				Quantification (%MNs)	
		Globular	Skein-like	Punctate	Diffuse	Incl./diff	Nuclear
1	Age-matched control	NA	NA	NA	NA	NA	NA
2	Age-matched control	NA	NA	NA	NA	NA	NA
3	Age-matched control	NA	NA	NA	NA	NA	NA
4	Age-matched control	−	−	−	−	−	+
5	Age-matched control	−	−	−	−	−	+
6	Age-matched control	−	−	−	√	+	+
7	Age-matched control	−	−	−	−	−	+
8	Age-matched control	−	−	−	√	+	+
9	Age-matched control	−	−	−	−	−	+
10	Age-matched control	√	√	−	√	+	+
11	fALS (SOD1, I113T)	√	−	√	√	++++	−
12	fALS (SOD1, I113T)	−	−	√	−	+++	−
13	fALS (SOD1, D101G)	√	−	√	−	++++	−
14	fALS (C9ORF72, 30+positive)	√	√	−	√	++++	−
15	fALS (unknown mutation)	√	√	−	√	++++	−
16	fALS (C9ORF72, 30+positive)	√	√	−	√	++++	+
17	fALS (C9ORF72, 30+positive)	√	√	√	√	+++	−
18	sALS	√	−	√	−	++++	−
19	sALS	√	−	−	−	++++	−
20	sALS	√	√	−	√	++	−
21	sALS	√	√	−	√	+++	−
22	sALS	NA	NA	NA	NA	NA	NA
23	sALS	√	√	−	√	+++	−
24	sALS	√	√	−	√	++++	−
25	sALS	√	√	√	√	+++	−
26	sALS	NA	NA	NA	NA	NA	NA

Fixed tissues were not available (NA) for some cases. The four morphologies of p62 pathology were noted as present (√) or absent (−), and the proportion of motor neurons exhibiting any of the four morphologies quantified. The proportion of motor neurons exhibiting nuclear p62 immunostaining was also quantified. Quantification classifications; − = 0%, + =  > 0–25%, ++26–50%, +++ = 51–75%, ++++ = 76–100%

Mechanistically, the accumulation and deposition of p62 in the cytoplasm is associated with a down-regulation of nucleocytoplasmic p62 shuttling in cultured non-neuronal cells [35]. This mechanism is also likely to be present in patients as nuclear p62 immunoreactivity was absent in spinal cord motor neurons of all but one ALS case, but was observed within up to 24% of control motor neurons (Fig. 3a, c; Table 2). As further evidence to this effect, the proportion of motor neurons exhibiting nuclear p62 and cytoplasmic p62 pathology were strongly inversely correlated (Fig. 2).

### A spectrum of overlapping disSOD1, pTDP-43 and p62 pathologies exist in post-mortem ALS spinal cord motor neurons

Given the apparent relationships between the presence of disSOD1 [47], TDP-43 and p62 pathologies in motor neurons in post-mortem ALS spinal cord tissues, we then employed multiplexed immunofluorescence microscopy to evaluate potential spatial interactions between these pathologies. Between 84 and 123 individual motor neurons exhibiting evidence of cytoplasmic disordered SOD1, pTDP-43 or p62 pathology were imaged in each of the three ALS subgroups, noting the presence of spatial overlap (colocalization) or segregation (coincidence) between pathologies where multiple were present within an imaged motor neuron.

**Fig. 4:**
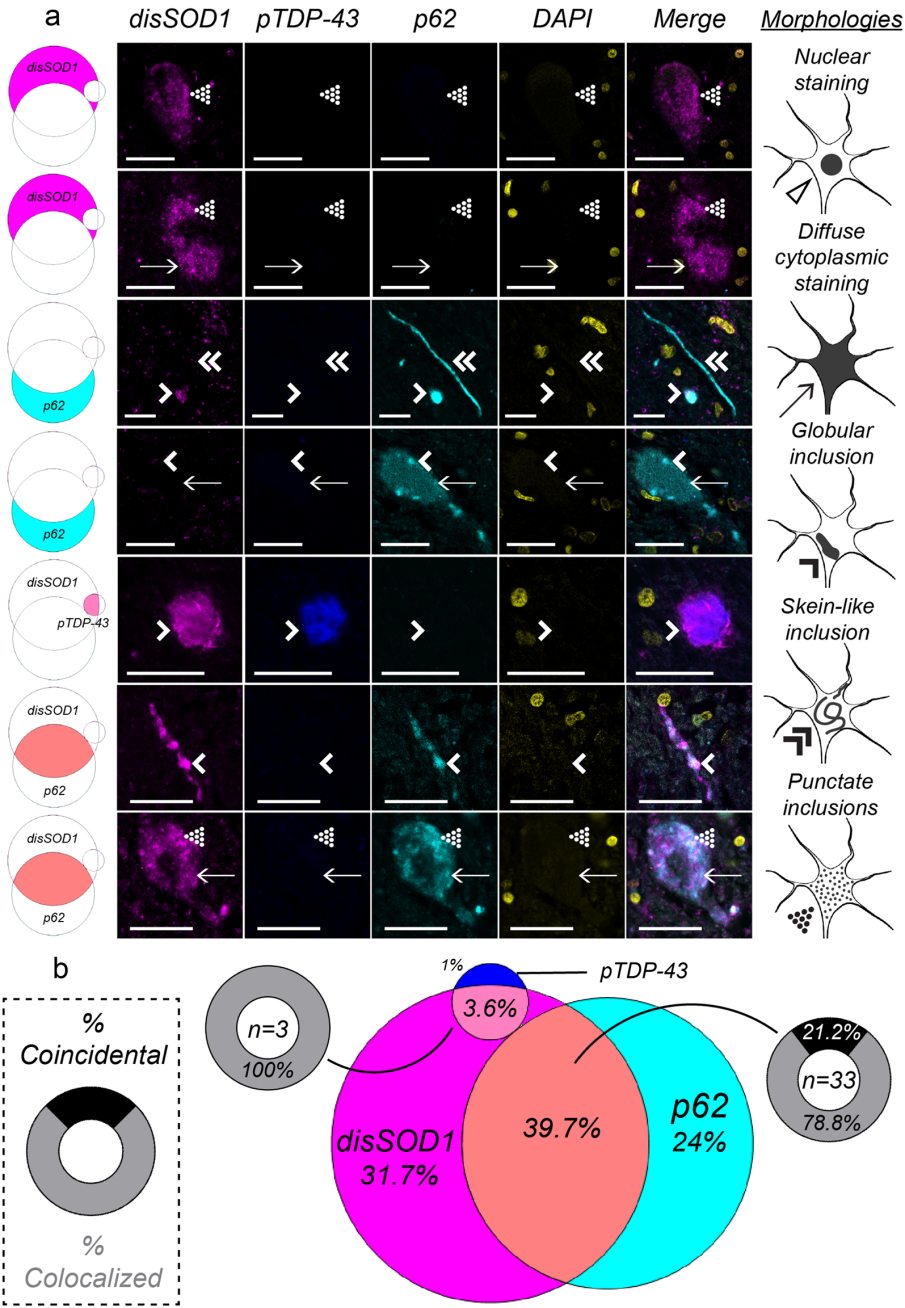
Coincidence and colocalization of disSOD1, pTDP-43 and p62 pathologies in spinal cord motor neurons of *SOD1*-fALS cases. **a** Immunostaining for disordered SOD1 (disSOD1), phosphorylated TDP-43 (pTDP-43) and p62 proteins using fluorescence microscopy in ventral spinal cord motor neurons of *SOD1*-fALS cases. We identified diffuse cytoplasmic staining (arrows), as well as punctate (dotted arrowheads), globular (single downwards arrowheads) and fibrillar skein-like (double downwards arrowheads) inclusions, comprised of one or more proteins of interest in these neurons. Rows represent each of the different combinations of pathologies observed in motor neurons of that subgroup; disSOD1 (magenta), pTDP-43 (blue) or p62 (cyan) deposition alone, disSOD1 and pTDP-43 (light pink), disSOD1 and p62 (orange), or pTDP-43 and p62 (light blue) co-deposition, or the co-deposition of all three pathologies (white). Scale bars in represent 25 µm. Antibody details are listed in Additional file 1: Table S3. No immunostaining was observed in spinal cord tissue sections processed as above in the absence of primary antibodies (Additional file 1: Figure S1). **b** A venn diagram illustrating the proportion of the 84 total motor neurons examined which exhibit each of the different individual or combined pathologies. Each segment corresponds to a row in panel **a**, and are pictured next to their corresponding row. Where co-deposition of two pathologies was observed within a motor neuron, we noted the presence (colocalized, grey) or absence (coincidental, black) of spatial overlap

**Fig. 5:**
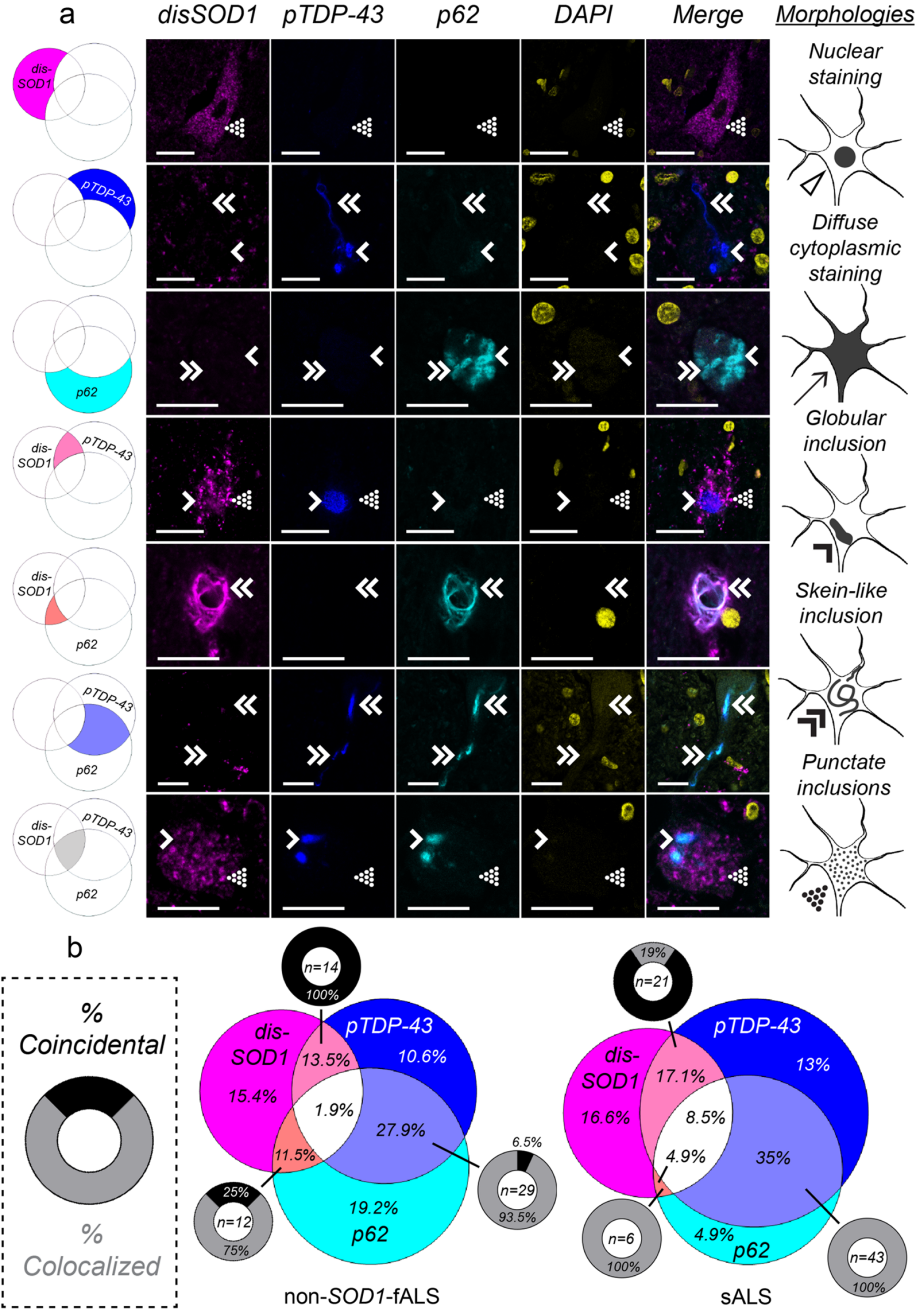
Coincidence and colocalization of disSOD1, pTDP-43 and p62 pathologies in spinal cord motor neurons of non-*SOD1*-fALS and sALS cases. **a** Immunostaining for disordered SOD1 (disSOD1), phosphorylated TDP-43 (pTDP-43) and p62 proteins using fluorescence microscopy in ventral spinal cord motor neurons of non-*SOD1*-fALS and sALS cases. We identified diffuse cytoplasmic staining (arrows), as well as punctate (dotted arrowheads), globular (single downwards arrowheads) and fibrillar skein-like (double downwards arrowheads) inclusions, comprised of one or more proteins of interest in these neurons. Rows represent each of the different combinations of pathologies observed in motor neurons of that subgroup; disSOD1 (magenta), pTDP-43 (blue) or p62 (cyan) deposition alone, disSOD1 and pTDP-43 (light pink), disSOD1 and p62 (orange), or pTDP-43 and p62 (light blue) co-deposition, or the co-deposition of all three pathologies (white). Scale bars in represent 25 µm. Antibody details are listed in Additional file 1: Table S3. No immunostaining was observed in spinal cord tissue sections processed as above in the absence of primary antibodies (Additional file 1: Figure S1). b A venn diagram illustrating the proportion of the total motor neurons examined (non-*SOD1*-fALS, *n* = 104; sALS, *n* = 123) which exhibit each of the different individual or combined pathologies. Each segment corresponds to a row in panel **a**, and are pictured next to their corresponding row. Where co-deposition of two pathologies was observed within a motor neuron, we noted the presence (colocalized, grey) or absence (coincidental, black) of spatial overlap

Disordered SOD1 pathology was present within a significant proportion of *SOD1*-fALS (75%), non-*SOD1*-fALS (42%) and sALS (47%) spinal cord motor neurons examined. Although pathological TDP-43 and mutant SOD1 exhibit a strong bilateral cross-seeding relationship in vitro [23, 36, 50], pathological TDP-43 was rarely (4%) present within spinal cord motor neurons of *SOD1*-fALS cases exhibiting disordered mutant SOD1 pathology (Fig. 4), in agreement with previous studies [25]. In contrast to *SOD1*-fALS cases, pTDP-43 pathology was present within 38–64% of neurons exhibiting wild-type disSOD1 pathology in non-*SOD1*-fALS and sALS cases, and 29–35% of pTDP-43-containing neurons possessed wild-type disSOD1 pathology (Fig. 5). Considering disordered wild-type SOD1 and pTDP-43 pathologies were primarily coincidental within these motor neurons (Fig. 5b), it is unlikely that they exhibit direct cross-seeding behaviour, suggesting alternative common mediators promote the development of both pathologies in these sub-groups.

Consistent with a role for p62 in binding mutant SOD1 and targeting it for clearance [17, 18], p62 was present within 79% of *SOD1*-fALS motor neurons exhibiting disordered mutant SOD1 pathology, and was colocalized with disordered mutant SOD1 pathology in 78% of these neurons (Fig. 4). No overlap was observed between p62 and pTDP-43 pathologies in *SOD1*-fALS cases, and therefore motor neurons exhibiting p62 pathology in the absence of mutant disSOD1 (24% of total surveyed population) may reflect efforts by p62 to promote the clearance of additional ALS-linked proteinopathies not examined in this study, including FUS [27]. In contrast to *SOD1*-fALS cases, p62 was only present within 13–17% of spinal cord motor neurons exhibiting disordered wild-type SOD1 pathology in non-*SOD1*-fALS and sALS cases (Fig. 5), reinforcing existing data demonstrating higher selectivity of p62 for mutant forms of the protein [17, 18]. Colocalization, rather than coincidence, of the two pathologies within the vast majority (75–100%) of this sub-population of non-*SOD1*-fALS and sALS motor neurons does, however, suggest a direct protein–protein interaction between p62 and wild-type SOD1.

As well as promoting the clearance of mutant SOD1 protein, p62 directly interacts with TDP-43 [46] and is reported to mitigate TDP-43 aggregation in an autophagy- and proteasome-dependent manner [5]. Despite its rarity in *SOD1*-fALS, pTDP-43 deposition was identified within a substantial proportion of non-*SOD1*-fALS (54%) and sALS (74%) spinal cord motor neurons examined in this study (Fig. 5). Consistent with previous studies conducted in post-mortem spinal cord tissues from sALS patients [11], 48–52% of pTDP-43-containing neurons exhibited p62 co-deposition in non-*SOD1*-fALS and sALS cases. These two pathologies were almost always (93.5–100%) colocalized, confirming a likely role for p62 in pathological TDP-43 clearance in vivo.

## Discussion

This study constitutes the first examination of SOD1, TDP-43 and p62 co-deposition in post-mortem ventral spinal cord tissues from *SOD1*-fALS, non-*SOD1*-fALS and sALS patients. We identified changes in the abundance of each pathology individually, as well as in the spatial overlap and coincidence of the three pathologies, within vulnerable spinal cord motor neurons between our three ALS subgroups. Aligning with existing data [14, 15, 25], our findings reveal disparate SOD1, TDP-43 and p62 aggregation profiles between the three ALS subgroups. Whilst our data largely support findings obtained from cellular and animal models of ALS, we identified apparent discrepancies in relationships between the three pathologies observed in ALS cases, compared with those reported in models of this disorder. While disease models constitute invaluable tools for interrogating relationships between disease pathologies, our study highlights the importance of contextualizing conclusions obtained from these models using corresponding data obtained from post-mortem patient tissues.

All ALS-associated *SOD1* mutations enhance the protein’s aggregation propensity [48], thus it is unsurprising that disordered mutant SOD1 protein constituted the dominant proteinopathy within motor neurons of *SOD1*-fALS cases in this study (Fig. 4). Significant mislocalization of disordered mutant SOD1 from the ER-golgi network to the cytoplasm accompanied SOD1 deposition within these neurons (related manuscript [47]), which we speculate may arise following inhibition of ER-golgi trafficking by mutant SOD1 itself [2]. Considering that disruption of protein transport along the ER-golgi axis constitutes an early event in *SOD1*-fALS pathogenesis [30], preceding protein aggregation [2], we propose that soluble nascent mutant SOD1, not aggregated mutant SOD1, may disrupt ER-golgi trafficking to promote cytoplasmic mutant SOD1 accumulation and deposition in motor neurons of *SOD1*-fALS cases. In addition to inhibiting ER-golgi transport, mutant SOD1 pathology is known to induce TDP-43 deposition in NSC-34 cells [50], and TDP-43 phosphorylation and C-terminal fragmentation in transgenic mice [23]. We, and others [25], however, found the presence of pTDP-43 pathology within motor neurons of *SOD1*-fALS cases to be extremely rare compared with abundant pTDP-43 pathology in motor neurons of other ALS subgroups (Figs. 4, 5). In addition to corroborating reports of a degree of phenotypic incongruence between common cellular and animal models of *SOD1*-fALS and human disease patients [39], our findings highlight the importance of recognizing that two proteins which possess the ability to interact in vitro may not necessarily participate in the same interaction in ALS patients. It must be acknowledged, however, that our data examines aggregated forms of both proteins, and does not inform on the likelihood of an interaction between soluble forms of these proteins in patients in vivo. In contrast to TDP-43 deposition, our data support a role for mutant SOD1 in inducing nucleocytoplasmic TDP-43 mislocalization (Figs. 1, 2), previously reported in NSC-34 cells [50]. The presence of TDP-43 mislocalization in the absence of overt cytoplasmic pTDP-43 pathology is strongly associated with neuronal toxicity in vitro [44], suggesting restoration of nuclear TDP-43, perhaps in combination with anti-aggregation therapies targeting disordered SOD1, may provide therapeutic benefit to *SOD1*-fALS patients.

Although substantial differences in aggregation profiles of SOD1, TDP-43 and p62 in *SOD1*-fALS, non-*SOD1*-fALS and sALS cases imply distinct pathways drive neurodegeneration in each ALS subgroup [11], the presence of common pathologies across subgroups indicates some pathways may be common to all patients, irrespective of the presence or absence of key gene mutations. Disordered SOD1 mislocalization and deposition are primarily attributed to *SOD1* gene mutations, and yet their presence in almost half of all spinal cord motor neurons in non-*SOD1*-fALS and sALS cases highlights the presence of alternative factors which promote SOD1 pathology in these cases [47, 48]. Altered post-translational modification of wild-type SOD1 can result in structural disorder, catalytic dysfunction and deposition of the protein in vitro and in vivo [16, 48], conferring biochemical properties that mirror the most unstable of mutant proteins [19]. Consequently, we speculate that disordered wild-type SOD1 can induce ER-golgi dysfunction in NSC-34 cells through a similar mechanism to mutant SOD1 [45], which may contribute to the cytoplasmic accumulation of disordered wild-type SOD1 pathology in these cells [29]. We propose that these data suggest that, like mutant SOD1 in *SOD1*-fALS motor neurons, disordered wild-type SOD1 may promote its own mislocalization and deposition in motor neurons of non-*SOD1*-fALS and sALS patients. Independent of alterations to SOD1 per se, *C9ORF72* hexanucleotide expansions are associated with elevated ER stress in induced pluripotent stem cells derived from *C9ORF72*-linked fALS patients [10] and pTDP-43 similarly inhibits ER-golgi transport in cultured Neuro2a cells [41]. We speculate that these data imply potential indirect contributions of these factors to wild-type SOD1 pathology in spinal cord motor neurons of non-*SOD1*-fALS and sALS cases in this study. Indeed, transfection-mediated overexpression of mutant and wildtype TDP-43 induces the accumulation of misfolded human wild-type SOD1 in cultured cell lines, as well as in primary spinal cord cells derived from human wild-type SOD1 transgenic mice [36]. Although such an association could be attributed to a direct cross-seeding interaction between these proteins in cases with *SOD1* mutations, wild-type SOD1 and TDP-43 do not interact in cultured motor neuron-like cells [23], and negligible spatial overlap was observed between SOD1 and TDP-43 pathologies within non-*SOD1*-fALS and sALS motor neurons in this study (Fig. 5). Collectively, we propose that these data suggest pathological TDP-43 may promote disordered wild-type SOD1 pathology through an indirect mechanism, such as impaired ER-golgi trafficking. Irrelevant of the underlying cause, we speculate that impaired ER-golgi trafficking may promote cytoplasmic disordered wild-type SOD1 accumulation in motor neurons of non-*SOD1*-fALS and sALS patients [29], constituting a common pathway leading to neurotoxic cytoplasmic SOD1 pathology in all ALS subgroups examined in this study.

Whilst wild-type and mutant TDP-43 induce wild-type SOD1 pathology in vitro [36], there have been no investigations into whether disordered wild-type SOD1 reciprocally induces TDP-43 mislocalization or deposition. Given mutant SOD1 induces TDP-43 pathology [23, 50], and disordered wild-type SOD1 adopts similar conformations to mutant SOD1 [19], it is certainly plausible that disordered wild-type SOD1 may also induce TDP-43 pathology. Indeed, wild-type SOD1 pathology impairs ER-golgi trafficking [41, 45], UPS function [13], and autophagy [1, 22] through similar mechanisms to mutant SOD1, which may reduce the clearance of pathological TDP-43 to result in its cytoplasmic accumulation. Further investigations are warranted to dissect the complex relationship between these pathologies, which may identify common and tractable therapeutic targets capable of mitigating the progression of both pathologies simultaneously.

Current therapies targeting disordered SOD1 and pathological TDP-43 in ALS patients primarily aim to reduce expression levels [31], stabilize protein structure [3, 38, 49], correct protein mislocalization [6] or enhance clearance mechanisms [9]. Included in the latter of these approaches are a number of pharmacological inducers of autophagy, many of which have already completed (tamoxifen [8], ClinicalTrials.gov NCT02166944), are currently ongoing (ibudilast [9], ClinicalTrials.gov NCT04057898; rapamycin [26], ClinicalTrials.gov NCT03359538) or are scheduled to enter (trehalose [7]) clinical trials for ALS. Our findings support the development of such interventions by demonstrating an interaction between mutant SOD1 and p62 within motor neurons of SOD1-fALS cases (Fig. 4), previously only identified in vitro, and between wild-type SOD1, TDP-43 and p62 in non-*SOD1*-fALS and sALS motor neurons (Fig. 5). To our knowledge, we are the first to report this interaction between wild-type SOD1 and p62. *SOD1* mutations exacerbate underlying structural flexibility within disordered SOD1 protein, providing a larger solvent-accessible surface area available for protein–protein interactions or post-translational modifications such as ubiquitin [48]. We speculate this may underlie the increased interaction between p62 and mutant SOD1, compared with wild-type SOD1, in our ALS cases; namely 4- and 8-fold increases in the spatial overlap between disordered SOD1 and p62 pathologies in motor neurons of SOD1-fALS cases compared with non-*SOD1*-fALS and sALS cases, respectively. Whilst our data support the development of therapies which enhance p62-mediated autophagy of disSOD1 and pathological TDP-43 in all forms of ALS, it must be acknowledged that this relies on the functionality of downstream protein quality control machinery; the proteasome and autophagosome, which become increasingly overwhelmed as ALS progresses [30]. Further, both knockout [20] and overexpression [32] of p62 accelerates motor neuron degeneration and the accumulation of ubiquitin-positive aggregates in spinal cord motor neurons of the same transgenic mutant SOD1 mouse strain, highlighting the need to regulate p62 levels and function within a tight range to achieve therapeutic benefit.

This study is the first to identify multiple relationships between disordered SOD1, pTDP-43 and p62 pathologies in spinal cord motor neurons of SOD1-fALS, non-SOD1-fALS and sALS cases, previously only studied in vitro. Comprehensive characterization of the relationships between these pathologies in all forms of ALS may advance our understanding of the multifaceted molecular pathways driving motor neuron death in this disorder, and identify potential therapeutic targets whose manipulation may slow or halt the development of damaging co-pathologies. We propose that investigation of novel therapies should optimally utilize cellular or animal model systems which more accurately express the range of ALS-linked co-pathologies identified in this study.

## Supplementary Information

**Additional file 1: Table S1.** Demographic and clinical information for human post-mortem tissue cases. **Table S2.** Demographic statistics for diagnostic groups. **Table S3.** Primary antibody details and applications. **Table S4.** Number of spinal cord motor neurons examined across all cases of each diagnostic group for quantification of each pathology of interest. **Fig. S1.** No primary controls and spectral validation of fluorescent microscopy workflow.

## Data Availability

The data and materials that support the findings of this study are available from the corresponding authors upon reasonable request.

## Abbreviations

ALS: Amyotrophic lateral sclerosis
C9ORF72: Chromosome 9 open reading frame 72
disSOD1: Structurally-disordered SOD1
ER: Endoplasmic reticulum
fALS: Familial amyotrophic lateral sclerosis
p62: Ubiquitin-binding protein 62/sequestosome 1
sALS: Sporadic amyotrophic lateral sclerosis
SOD1: Superoxide dismutase-1
TDP-43: TAR DNA-binding protein 43
