# Supplementary material for: Co-deposition of SOD1, TDP-43 and p62 proteinopathies in ALS: evidence for multifaceted pathways underlying neurodegeneration
Source: Acta Neuropathol Commun. 2022 Aug 25;10:122. doi: 10.1186/s40478-022-01421-9 (PMC9404564; doi:10.1186/s40478-022-01421-9)
Supplement: Supplementary file 1 — Additional file 1: Table S1. Demographic and clinical information for human post-mortem tissue cases. Table S2. Demographic statistics for diagnostic groups. Table S3. Primary antibody details and applications. Table S4. Number of spinal cord motor neurons examined across all cases of each diagnostic group for quantification of each pathology of interest. Fig. S1. No primary controls and spectral validation of fluorescent microscopy workflow. [file 40478_2022_1421_MOESM1_ESM.docx]

**Supplementary Information**

***Inventory of Supplementary Information***

- Supplementary Tables 1-4

1. Demographic and clinical information for human post-mortem tissue cases.
2. Demographic statistics for diagnostic groups.
3. Primary antibody details and applications.
4. Number of spinal cord motor neurons examined across all cases of each diagnostic group for quantification of each pathology of interest.

- Supplementary Figure 1
  - No primary controls and spectral validation of fluorescent microscopy workflow.

**Supplementary Table 1. Demographic and clinical information for human post-mortem tissue cases.**

| Case # | Diagnostic Group | Age | Sex | PMI | Site of onset | Cause of Death | Fixed Tissue Regions | Fresh Frozen Tissue Regions |
| --- | --- | --- | --- | --- | --- | --- | --- | --- |
| 1 | Age-matched Control | 51 | F | 33 | N/A | Lung Cancer | TSpC | TSpC, OCx |
| 2 | Age-matched Control | 54 | M | 30 | N/A | Cancer | TSpC | TSpC, OCx |
| 3 | Age-matched Control | 73 | F | 27 | N/A | NA | TSpC | TSpC, OCx |
| 4 | Age-matched Control | 65 | M | 25 | N/A | Ruptured Aortic Aneurysm | TSpC, CSpC | TSpC, CSpC, OCx |
| 5 | Age-matched Control | 50 | M | 24 | N/A | Atherosclerotic Cardiovascular Disease | TSpC | TSpC, CSpC, OCx |
| 6 | Age-matched Control | 48 | M | 22 | N/A | Hypertensive Atherosclerosis Heart Disease | TSpC, CSpC | TSpC, CSpC, OCx |
| 7 | Age-matched Control | 49 | F | 26 | N/A | Hypertensive Atherosclerosis Heart Disease | TSpC | TSpC, CSpC, OCx |
| 8 | Age-matched Control | 55 | M | 23 | N/A | Cardiac Arrythmia (Cardiomegaly) | TSpC, CSpC | TSpC, CSpC, OCx |
| 9 | Age-matched Control | 49 | M | 22 | N/A | Cardiac Tamponade | TSpC, CSpC | TSpC, CSpC, OCx |
| 10 | Age-matched Control | 50 | M | 22 | N/A | Hypertensive Atherosclerosis Heart Disease | TSpC, CSpC | TSpC, CSpC, OCx |
| 11 | fALS (SOD1, I113T) | 47 | M | 13.5 | Bulbar | Complication of Disorder | TSpC | TSpC, OCx, OCx |
| 12 | fALS (SOD1, I113T) | 70 | F | 56 | Bulbar | Complication of Disorder | TSpC | TSpC, OCx, OCx |
| 13 | fALS (SOD1, D101G) | 46 | F | 5 | Limb - upper | Complication of Disorder | TSpC | TSpC, OCx, OCx |
| 14 | fALS (C9ORF72, 30+ positive) | 64 | M | 68 | Bulbar | Complication of Disorder | TSpC | TSpC, OCx |
| 15 | fALS (C9ORF72, 30+ positive) | 62 | M | 20 | Bulbar | Complication of Disorder | TSpC, CSpC | TSpC, CSpC, OCx |
| 16 | fALS (C9ORF72, 30+ positive) | 55 | M | 24 | Limb - upper (right) | Complication of disorder | TSpC, CSpC | TSpC, CSpC, OCx |
| 17 | fALS (unknown mutation) | 65 | M | 7 | Limb - lower (left) | Complication of Disorder | TSpC, CSpC | TSpC, CSpC, OCx |
| 18 | sALS | 70 | F | 15 | Limb - upper | Complication of Disorder | TSpC | TSpC, OCx |
| 19 | sALS | 50 | F | 56 | Limb - lower | Complication of Disorder | TSpC | TSpC, OCx |
| 20 | sALS | 68 | F | 17 | Limb - lower | Complication of Disorder | TSpC | TSpC, CSpC, OCx |
| 21 | sALS | 54 | F | 12 | Limb - upper and lower | Complication of Disorder | TSpC, CSpC | TSpC, CSpC, OCx |
| 22 | sALS | 56 | M | 22 | Limb - lower | Complication of Disorder | NA | TSpC, CSpC, OCx |
| 23 | sALS | 68 | M | 4 | Limb - upper and lower | Complication of Disorder | TSpC, CSpC | TSpC, CSpC, OCx |
| 24 | sALS | 57 | F | 20 | Limb - lower (right) | Complication of Disorder | TSpC, CSpC | TSpC, CSpC, OCx |
| 25 | sALS | 62 | M | 4 | Bulbar | Complication of Disorder | TSpC, CSpC | TSpC, CSpC, OCx |
| 26 | sALS | 70 | F | 29 | Limb - lower | Complication of Disorder | NA | TSpC, CSpC, OCx |

**Abbreviations;** CSpC, cervical spinal cord; fALS, familial amyotrophic lateral sclerosis; F, female; M, male; N/A, not applicable; NA, not available; OCx, occipital cortex; PMI, post-mortem interval; sALS, sporadic amyotrophic lateral sclerosis; TSpC, thoracic spinal cord.

**Supplementary Table 2. Demographic statistics for diagnostic groups.**

| *Variable* | *Diagnostic group* | | | | *Significantly different?* |
| --- | --- | --- | --- | --- | --- |
|  | *Control* | *SOD1-fALS* | *Non-SOD1-fALS* | *sALS* |  |
| *n* | 10 | 3 | 4 | 9 | N/A |
| *Sex (M:F)* | 7:3 | 1:2 | 4:0 | 3:6 | CD^a^ |
| *Age (years)*  *[range]* | 54.4±2.6  [48-73] | 54.3±7.8  [46-70] | 61.5±2.3  [55-65] | 61.67±2.6 [50-70] | N.S. (*p* = 0.14)^b^ |
| *PMI (hrs)* | 25.4±1.2 | 24.8±15.8 | 29.8±13.3 | 19.9±5.3 | N.S. (*p* = 0.23)^b^ |

**Footnotes;** ^a^ Chi-square test, ^b^ Kruskal-Wallis test

**Abbreviations;** CD, could not determine; fALS, familial amyotrophic lateral sclerosis; F, female; M, male; N/A, not applicable; N.S., not significant; PMI, post-mortem interval; sALS, sporadic amyotrophic lateral sclerosis

**Supplementary Table 3.** **Primary antibody details and applications.**

| **Antibody** | **Source (cat#)** | **Class** | **Host** | **Species reactivity** | **Immunogen** | **Dilution** |
| --- | --- | --- | --- | --- | --- | --- |
| DisSOD1 (UβB) | StressMarq Biosciences, British Columbia, Canada (SPC-205) | P | Rb | Hu, Ms, Rat | N-terminal region, SOD1 protein with unfolded beta barrel | 1:200 |
| TDP-43 | Abcam, Cambridge, UK (ab154047) | P | Rb | Hu, Ms, Rat | Recombinant fragment corresponding to amino acids 1-289 of human TDP43 | 1:300 |
| pTDP43 | Cosmo Bio Co, Tokyo, Japan (TIP-PTD-M01) | M | Ms | Hu | Phosphorylated C-terminal TDP-43 peptide (Ser409/410) | 1:500 |
| p62 | BD Transduction, Frankin Lakes, NJ, USA (610833) | M | Ms | Hu | Amino acids 257-437 of human p62 lck ligand | 1:300 |

**Abbreviations**; DisSOD1, structurally-disordered superoxide dismutase 1; Hu, human; M, monoclonal; Ms, mouse; p62, p62 lck ligand/zeta-interacting protein/SQSTM1; P, polyclonal; Rb, rabbit; TDP-43, TAR DNA-binding protein 43.

**Supplementary Table 4.** **Number of spinal cord motor neurons examined across all cases of each diagnostic group for quantification of each pathology of interest.**

| **Pathology(ies)** | **Immunolabel antibody(ies)** | **Stain type** | **Total number of spinal cord motor neurons examined** | | | |
| --- | --- | --- | --- | --- | --- | --- |
|  |  |  | **Control** | ***SOD1*-fALS** | **Non-*SOD1*-fALS** | **sALS** |
| Nuclear TDP-43 | Abcam, Cambridge, UK (ab154047) | DAB | 2048 | 58 | 146 | 370 |
| Phosphorylated TDP-43 pathology | Cosmo Bio Co, Tokyo, Japan (TIP-PTD-M01) | DAB | 2097 | 57 | 144 | 376 |
| Nuclear p62, cytosolic p62 pathology | BD Transduction, Frankin Lakes, NJ, USA (610833) | DAB | 1999 | 57 | 150 | 370 |
| DisSOD1* | StressMarq Biosciences, British Columbia, Canada (SPC-205) | DAB | 2001 | 53 | 152 | 363 |
| DisSOD1, pTDP-43 and p62 colocalization | StressMarq Biosciences (SPC-205), Cosmo Bio Co (TIP-PTD-M01), BD Transduction (610833) | IF | N/A | 84 | 104 | 123 |

**Abbreviations**; DAB, 3,3′-Diaminobenzidine; DisSOD1, structurally-disordered superoxide dismutase 1; fALS, familial amyotrophic lateral sclerosis; IF, immunofluorescence; p62, p62 lck ligand/zeta-interacting protein/SQSTM1; sALS, sporadic amyotrophic lateral sclerosis; TDP-43, TAR DNA-binding protein 43.

*****quantification data presented in related manuscript [47].


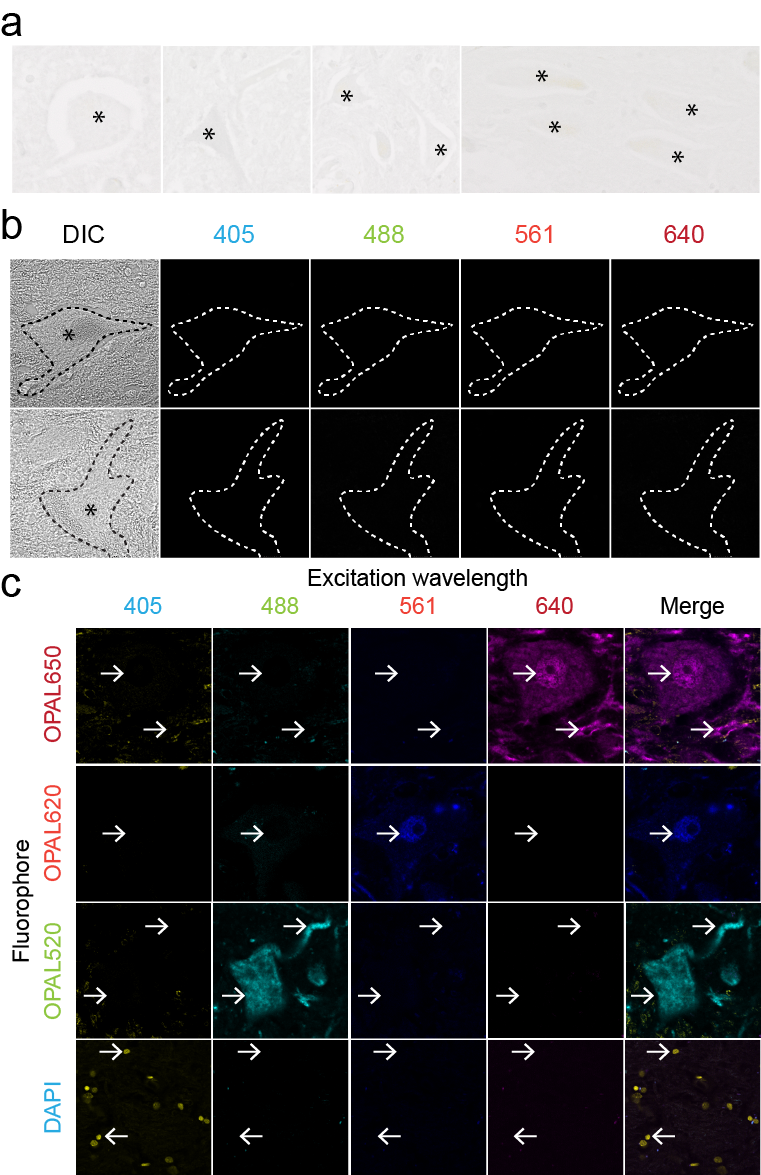


**Supplementary Figure 1**. **No primary controls and spectral validation of fluorescent microscopy workflow.** **a,** **b** Characterisation of non-specific binding of secondary antibodies and either DAB chromagen (**a**) or OPAL fluorophores (**b**) was performed using control tissue sections processed in the absence of primary antibodies. Negligible DAB staining was observed upon imaging of tissues incubated with HRP-conjugated secondary antibodies and DAB chromagen, indicating minimal non-specific binding of secondary antibodies or DAB to tissues. Minimal fluorescence was observed upon exposure of tissues to characteristic OPAL fluorophore excitation wavelengths, indicating minimal non-specific binding of secondary antibodies and OPAL fluorophores to tissues. Neurons are marked with asterisks in **a** and **b**. **c** To validate the absence of spectral overlap between our employed fluorophores at the confocal microscope acquisition settings employed in this study, tissue sections were labelled with TUJ-1 primary antibody (Biolegend, USA) and an anti-mouse HRP-conjugated secondary antibody, and were then labelled with individual OPAL fluorophores (OPAL650, OPAL620, OPAL520) or DAPI. Images were then captured at all four characteristic fluorophore wavelengths for each individually stained section using sequential acquisition, where fluorescent excitation and image acquisition are performed for each channel separately. This minimizes spectral bleed through between channels, as spectral emissions from one fluorophore cease prior to excitation and acquisition for the next channel. **b** and **c** have been reported previously [47].
